# Lower-Limb Muscle Impairments in Patients with COPD: An Overview of the Past Decade

**DOI:** 10.3390/cells15030220

**Published:** 2026-01-23

**Authors:** Bente Brauwers, Martijn A. Spruit, Frits M. E. Franssen, Anouk W. Vaes, Felipe V. C. Machado

**Affiliations:** 1 Department of Research and Development, Ciro, 6085 NM Horn, The Netherlands; 2 NUTRIM Research Institute of Nutrition and Translational Research in Metabolism, Faculty of Health, Medicine and Life Sciences, Maastricht University, 6229 HX Maastricht, The Netherlands; 3 Department of Respiratory Medicine, NUTRIM Research Institute of Nutrition and Translational Research in Metabolism, Maastricht University Medical Center+, 6229 HX Maastricht, The Netherlands; 4 Rehabilitation Research Center (REVAL), Faculty of Rehabilitation Sciences, Hasselt University, 3500 Diepenbeek, Belgium; 5 BIOMED-Biomedical Research Institute, Faculty of Medicine and Life Sciences, Hasselt University, 3500 Diepenbeek, Belgium

**Keywords:** lower-limb muscle dysfunction, COPD, mitochondrial dysfunction, oxidative damage, muscle protein synthesis, systemic inflammation, smoking, hypoxia, exercise training

## Abstract

Chronic obstructive pulmonary disease (COPD) is a chronic respiratory disease characterized by airflow limitation. Apart from airflow limitation, patients with COPD may also suffer from extra-pulmonary features such as lower limb muscle dysfunction that contribute to an impaired health status. Since the latest statement on lower-limb muscle dysfunction in COPD in 2014, substantial new evidence has emerged with regard to molecular, cellular, and functional mechanisms underlying muscle plasticity. Therefore, this review aims to provide an updated overview of molecular, cellular, and functional mechanisms of lower-limb muscle plasticity in COPD, integrating evidence that has emerged since the 2014 statement on lower limb muscle dysfunction. Additionally, the effects of exercise training on mechanisms of limb muscle dysfunction are explained. From the evidence of the last decade, it can be concluded that limb muscle dysfunction is a multifactorial process driven by both intrinsic alterations and impairments to the muscle as well as extra-pulmonary influences, thereby reinforcing the need for integrated therapeutic strategies.

## 1. Introduction

Chronic obstructive pulmonary disease (COPD) is a disabling chronic respiratory disease affecting millions of people around the world [1]. Besides the cardinal airflow limitation, patients with COPD may also suffer from extra-pulmonary and behavioural traits that contribute to an impaired health status [2].

A loss of lower-limb muscle mass and intramuscular abnormalities such as reduced mitochondrial function, dysregulated protein turnover, increased oxidative damage, and impaired muscular energetics are widely recognized as important extra-pulmonary traits in patients with COPD [3,4,5,6,7,8,9,10,11,12,13,14,15]. Indeed, patients’ exercise tolerance may, at least in part, be limited by these lower-limb defects [4,5,9,13,15]. Moreover, lower-limb muscle weakness and atrophy have been associated with early death [16]. Therefore, early diagnosis and treatment seem of high clinical relevance. The importance of lower-limb muscle defects in COPD is emphasized by the fact that international statements have been published over the years [17,18].

Since the latest statement on lower-limb muscle dysfunction in COPD in 2014 [18], substantial new evidence has emerged. Therefore, this narrative review addresses the research gaps as identified by Maltais and colleagues [18] that were most closely aligned with skeletal muscle plasticity with evidence from the last 10 years. Emphasis was placed on the main molecular mechanisms involved, their onset, as well as associated risk factors for muscle dysfunction and the potential for improvement through exercise training.

## 2. Gap 1: Molecular Mechanisms of Limb Muscle Dysfunction

One of the research gaps stated by Maltais et al. [18] was the need for more mechanistic work on cellular and molecular pathways, such as proteolysis and protein synthesis, mitochondrial biology, and oxidative stress.

**Figure 1:**
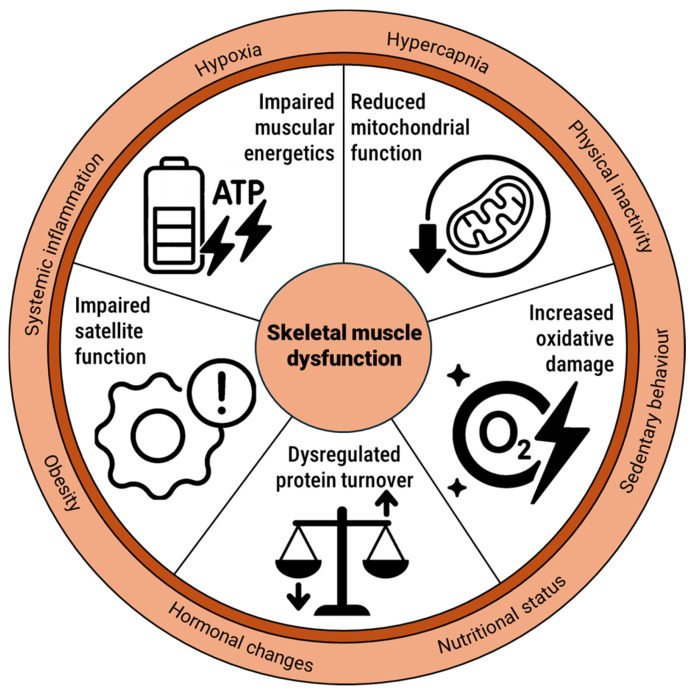
Overview of key cellular mechanisms leading to limb skeletal muscle dysfunction in COPD.

To advance our understanding of skeletal muscle dysfunction in COPD, it is essential to explore the biological processes that drive its development at the cellular level. In the following section, we delve into key mechanisms that are believed to contribute to muscle dysfunction in COPD. We focus on five interconnected pillars of muscle health: muscle protein synthesis, mitochondrial function, oxidative damage, muscular energetics, and satellite cell biology (Figure 1). Together, these mechanisms shape the muscle’s capacity to produce energy, maintain integrity, regenerate tissue, and adapt to physiological stress. By examining their roles, we aim to shed light on how cellular disturbances can ultimately translate into reduced muscle function.

### 2.1. Dysregulations in Protein Turnover

There are several intrinsic mechanisms that are believed to contribute to skeletal muscle dysfunction, such as muscle loss through catabolic activation and/or anabolic suppression and calcium desensitization. Several signals that are also hallmarked for COPD, such as smoking, malnutrition, immobilization, and in some cases hypoxia and/or hypercapnia, eventually lead to lower-limb muscle atrophy through accelerated intracellular protein degradation. This protein breakdown occurs through several pathways, including the ubiquitin–proteasome pathway, the lysosomal pathway, and the myostatin–Smad3 pathway [19]. Since the 2014 statement, several studies have investigated the involvement of markers in muscle protein breakdown. A cross-sectional study by Ito et al. [20] found that in the muscle of sarcopenic patients with COPD, there were significantly reduced Parkin concentrations and 2.5 times greater MuRF-1 protein levels compared to healthy age- and sex-matched controls, suggesting impaired Parkin-mediated mitophagy and increased muscle protein breakdown. An in vitro study investigating the effects of pro-oxidant treatments on oxidative stress levels and the atrophic signalling pathway of cultured COPD myotubes showed that COPD myotubes treated with H_2_O_2_ had increased ROS production and protein carbonylation. Additionally, it showed more pronounced myotubular atrophy caused by increased expression of MuRF-1 and FoxO1 when compared to healthy myotubes, demonstrating the involvement of oxidative stress in the atrophy of COPD muscle cells in vitro via the FoxO1/MuRF1/atrogin-1 signalling pathway of the ubiquitin/proteasome system [21]. In a another study analysing vastus lateralis muscle biopsies from patients with COPD, both with and without sarcopenia, molecular markers of protein degradation (FOXO1, LC3BII/I, and p-ULK1) and protein synthesis signalling (AKT1 and p-4E-BP1) were simultaneously upregulated when compared to non-sarcopenic controls, indicating heightened but unbalanced muscle protein turnover [22]. These alterations were even more pronounced in patients with COPD and sarcopenia, alongside increased expression of myogenic signalling (MYOG) and myostatin (MSTN), reflecting a paradoxical activation of both anabolic and catabolic pathways [22].

Another way to induce muscular atrophy is by modifying the myostatin–Smad3 pathway, which acts as a negative regulator of muscle growth. Different studies have shown that COPD cells present increased myostatin expression when compared to healthy controls [23,24,25] and non-sarcopenic patients with COPD [25], and reduced anabolic signalling and increased atrogin-1 when compared to healthy controls, indicating enhanced protein breakdown, altogether indicating a shift towards muscle atrophy and reduced muscle protein synthesis [23]. Núñez-Robainas et al. [25] reported that the myostatin/Smad2/Smad3 pathway is activated differently in sarcopenic patients with COPD when compared to patients with COPD without sarcopenia and healthy controls. These differences lie in an increased p-Smad2/smad2 ratio, downregulated Smad3 gene expression, and increased p-Smad3/Smad3 and Smad4 protein levels in the sarcopenic COPD group when compared to the other two groups. In sarcopenic patients with COPD, myostatin and p-Smad/Smad3 levels showed a negative correlation with the fat-free mass index [25]. Markers for muscle regeneration, such as Pax7, Myf5, MyoD, and MyHC-I, are significantly lower in COPD when compared to controls, suggesting impaired regenerative signalling [24]. In contrast, Thériault et al. [26] reported significant increases in MyoD and Myf5 in COPD with or without muscle atrophy, with no differences in Pax7 levels between the COPD groups (with and without atrophy) and healthy controls. Additionally, this study found reduced expression of later differentiation markers such as myogenin, MRF4, and MHC in COPD when compared to healthy subjects [26]. Another measure for regeneration is the number of central nuclei per 100 muscle fibres, which was lower in COPD patients with muscle atrophy when compared to patients with COPD with preserved muscle mass [26,27]. In summary, sarcopenic patients with COPD exhibit elevated markers of muscle protein breakdown and altered activation of the myostatin/Smad2/Smad3 pathway compared with non-sarcopenic patients and healthy controls. Findings on regenerative markers are somewhat mixed: some studies report reduced expression of regeneration-related proteins, suggesting impaired regenerative capacity, while others, such as Thériault et al. [26], observed increases in early myogenic markers but reductions in later differentiation markers and fewer central nuclei in atrophied muscle [26,27]. Lastly, MicroRNAs (miRs), small non-coding regulators of gene expression, have emerged as potential modulators of mitochondrial function and protein synthesis. A 2019 study [28] measured miR-542-3p and miR-542-5p in human skeletal muscle as part of its gene expression profile and found that these were overexpressed (~5–6-fold higher) in muscles of patients with COPD when compared to healthy age-matched controls, and this overexpression was linked to impaired protein synthesis and loss of physical performance in COPD [28]. These data collectively indicate dysregulation of both protein turnover and regenerative signalling in the skeletal muscle of patients with COPD. Whereas the 2014 ATS statement primarily identified MuRF-1 and atrogin-1-mediated proteolysis as key mechanisms, recent studies reveal a broader dysregulation of protein turnover and regenerative signalling involving myostatin/Smad pathways.

Decreased oxygen delivery to the muscles could also impact muscle protein turnover. To date, no studies have specifically investigated the effects of decreased oxygen delivery to the muscles on protein turnover in COPD, but studies have focused on lower-limb microcirculation in COPD. Indeed, Hartmann et al. [29] and Layec et al. [30] showed that at rest, leg blood flow, mean arterial pressure, and leg vascular conductance did not differ between COPD and age- and sex-matched controls [29] or healthy controls [30]. Both studies reported that patients with COPD demonstrated lower arterial oxygen tension, lower saturation, and lower mean capillary oxygen tension when compared with the control groups, indicating reduced microvascular oxygen availability at baseline. However, the COPD group did show higher resting skeletal muscle oxygen uptake and skeletal muscle oxygen conductance compared to control groups, suggesting a compensatory increase in muscle diffusive oxygen transport capacity [29,30]. These findings suggest that preserved muscle perfusion but reduced oxygen availability may limit mitochondrial ATP production and thereby contribute to dysregulated protein turnover. While the 2014 ATS statement primarily addressed impairments in muscle oxygen delivery and microcirculation in relation to exercise intolerance and oxidative capacity, it did not consider their potential role in regulating muscle protein turnover. The present findings extend this framework by suggesting that, despite preserved perfusion of the muscle, reduced oxygen availability may be a limiting factor in mitochondrial ATP production and thereby impair anabolic processes, contributing to dysregulated muscle protein turnover in COPD.

### 2.2. Mitochondrial Function in Limb Muscles of Patients with COPD

To illustrate how mitochondrial dysfunction manifests in COPD, recent studies have examined the functional oxidative capacity of muscles and markers of mitochondrial biogenesis and function. A study investigating muscular oxidative capacity using near-infrared spectroscopy to determine upper- and lower-limb muscle oxygen consumption of 19 patients with COPD and 19 age- and sex-matched participants with normal spirometry found that oxidative capacity was approximately ~20–30% lower in both upper- and lower-limb muscles of patients with COPD compared to age- and sex-matched controls, while resting tissue oxygenation was similar between groups [31]. With regard to mitochondrial DNA (mtDNA) copy numbers, a study found these were lower in COPD muscle fibres compared to healthy, age-matched controls, and there was a reduced mitochondrial transcription factor A (TFAM) protein-to-transcript ratio and citrate synthase activity, indicating impaired mitochondrial biogenesis, mitochondrial content, and function [32]. Another study found a lower ratio of mtDNA to total nuclear DNA in the vastus lateralis muscle of individuals with COPD, suggesting fewer mitochondria per cell compared to healthy controls [33]. These results indicate that reductions in oxidative capacity and impaired mitochondrial biogenesis, mitochondrial content, and function are an important physiological component of muscle dysfunction in COPD.

### 2.3. Oxidative Damage and Muscle Dysfunction

The ATS 2014 statement identified oxidative stress as a key driver of muscle dysfunction in COPD, with elevated reactive oxygen species (ROS) and/or reactive nitrogen species (RNS) markers in blood and limb muscles, even at rest, that were linked to reduced strength and exercise performance [18]. The precise contribution of ROS/RNS to muscle dysfunction was not fully understood; however, more recent research offers valuable updates. A study by Haji et al. [34] showed that ROS were elevated in the quadriceps muscle of GOLD (Global Initiative for Chronic Obstructive Lung Disease) grade 2–4 patients compared to healthy controls, and only in GOLD grade 3–4 patients was the antioxidant enzyme SOD2 reduced [34]. This study, however, did not show correlations between ROS levels and quadriceps strength, lung function, 6 min walk distance, or peak oxygen consumption. Additionally, when assessing oxidative damage markers in the vastus lateralis muscle of patients with COPD, the toxic byproducts of ROS, a doubling of 4-hydroxynonenal (HNE) and a 50% increase in 8-OHdG levels were found in COPD when compared to age-matched controls [32]. Another study also found increased HNE levels in COPD myotubes and found elevated carbonylation in COPD myoblasts and myotubes when compared to those of healthy individuals [23]. Similarly, malondialdehyde protein adducts—endogenous genotoxic products formed upon lipid peroxidation—were found to be increased at the muscular and systemic level in patients with COPD compared with sedentary healthy controls [35]. Konokhova et al. [32] reported that mtDNA deletions were more prevalent in COPD when compared to healthy controls and correlated with higher oxidative damage, longer smoking histories, and lower maximal oxygen consumption [32]. A study investigating antioxidant treatment with ascorbic acid to lower ROS in skeletal muscle cells of patients with COPD showed that it decreased the expression of autophagy markers such as LC3 and BNIP3, accompanied by increased myotube dynamometer compared to healthy controls [36]. These findings suggest that oxidative stress level contributes to the regulation of autophagy, a process involved in COPD myotube atrophy in vitro [36]. In summary, ROS and toxic byproducts were increased in both muscle and serum of patients with COPD compared to healthy controls, without correlations with exercise capacity. Oxidative stress appears to drive autophagy in skeletal muscle, contributing to myotube atrophy, while antioxidant treatment can reduce autophagy marker expression and improve muscle cell function. While the 2014 ATS statement identified increased oxidative stress as a contributor to muscle dysfunction, it lacked mechanistic insight into mitochondrial genomic integrity and autophagy. The more recent evidence summarized here extends this framework by linking oxidative stress to mitochondrial DNA damage, impaired mitochondrial biogenesis, and autophagy-driven muscular atrophy.

Guo et al. [37] observed increased numbers of autophagosomes, autophagy markers, autophagy-related genes, and oxidative stress markers in the lower-limb muscles of individuals with COPD compared to controls [37]. In addition, they found that higher lipofuscin inclusions (a marker for oxidative damage) correlated with autophagosome numbers, indicating that cells experiencing greater oxidative stress also show greater activation of autophagy. Regarding mitophagy, it was found that in COPD, Parkin levels increased, while FUNDC1 and PINK1 decreased at the protein level when compared to controls, suggesting enhanced clearance of damaged mitochondria in patients with COPD [33]. The same team investigated inflammatory status in two groups of patients with COPD: those with lower levels of C-reactive protein (CRP) and those with higher levels of CRP. In this study, patients with COPD and with high CRP had increased BNIP3 (a protein related to autophagy) at the protein level when compared to patients with COPD with lower levels of CRP. These results highlight that mitophagy is associated with systemic inflammation [38]. To conclude, lower-limb muscles in COPD showed increased autophagy and oxidative stress markers, disrupted mitophagy, and inflammation-driven changes in autophagy-related proteins. In contrast to the 2014 ATS statement that identified autophagy as an understudied pathway, this recent evidence demonstrates coordinated dysregulation of autophagy, mitophagy, and inflammation in COPD skeletal muscle.

### 2.4. Muscular Energetics: Low Mechanical Efficiency and Energy Expenditure

The 2014 ATS statement reported that individuals with COPD showed higher oxygen consumption and ATP cost during exercise, along with increased resting and total energy expenditure, likely reflecting reduced contractile efficiency and elevated protein turnover. Tényi et al. [39] showed downregulation of creatine kinases and substrates for creatine synthesis in COPD muscle compared to healthy sedentary controls, indicating impaired resting muscle energy production, which correlated with blood lactate and systemic inflammatory markers [39]. Another study examined specific mitochondrial hubs, such as IDH2 and SIRT1, and found these to be downregulated in COPD when compared to age- and sex-matched controls, indicating impaired tricarboxylic acid (TCA) cycle and oxidative phosphorylation capacity [40]. These findings suggest that COPD is associated with impaired muscle energy metabolism and mitochondrial function. While the 2014 ATS statement highlighted general mitochondrial dysfunction and altered bioenergetics in COPD, these recent studies provide new insights with regard to specific pathways involved in impaired muscular energy metabolism.

### 2.5. Satellite Cells in COPD

Satellite cells play a crucial role in muscle repair and regeneration, and a decline in these cells results in poorer muscle recovery and adaptation [41,42]. Several studies found reduced fusion capacity per myotube and a smaller myotube diameter [23], but no proliferation defects [23,24,26,27,43] in muscle biopsies from patients with COPD compared to healthy controls [23,24,26,27,43]. These data suggest that muscle regeneration is compromised as a result of impaired satellite cell function. Importantly, the impairment does not arise from an inability of the satellite cells to proliferate; rather, it stems from an impairment in the ability to fuse effectively. This fusion defect prevents the formation of robust, mature myofibres, which ultimately limits the restoration of the muscles. One study demonstrated the loss of quiescent satellite cells in patients with COPD and sarcopenia when compared to controls, but with compensatory activation of the remaining cells [24]. Lastly, a study investigating the potential relationship between muscle satellite cells and capillaries in COPD found that patients with COPD had lower capillarization compared with controls; however, capillary density per area did not differ between groups [44]. In addition, satellite cell numbers were similar when comparing COPD to controls, but satellite cells were located farther from capillaries in COPD in both fibre types, compared to age-, sex-, and BMI-matched controls [44]. This greater distance may contribute to the dysfunction of satellite cells observed in patients with COPD. To summarize, impaired fusion capacity of satellite cells in COPD contributes to disrupted muscle regeneration, and these cells are positioned farther from capillaries in both muscle fibre types. In the 2014 ATS statement, increased expression of myostatin and Nedd4 could influence satellite cell proliferation, speculating that muscle repair is likely altered in COPD. Indeed, the new evidence suggests that impaired fusion capacity of satellite cells contributes to disrupted muscle regeneration. Additionally, a new study pointed out that satellite cells lie farther away from capillaries.

## 3. Gap 2: Risk Factors for Muscle Dysfunction

### 3.1. Onset of Muscle Dysfunction in Smokers

It is known that smoking is one of the most important risk factors for COPD development [45]. Interestingly, cigarette smoke exposure is also a contributor to muscle dysfunction even before the onset of lung disease [46]. A 2019 systematic review [47] investigating the association between smoking and muscle dysfunction in smokers and patients with mild COPD showed contrasting results; on one hand, several studies reported lower upper- and lower-limb muscle strength in smokers when compared to non-smokers, while other studies found no differences between the groups. Notably, half of the studies reporting non-significant findings involved participants with shorter smoking histories, suggesting that cumulative exposure might be relevant. Evidence also suggests that muscle impairments in COPD may develop early and independently of ageing, as younger patients with early-onset severe COPD show body compositions comparable to older patients with similar disease severity [48].

Smoking leads to chronic exposure to nicotine, which activates nicotinic acetylcholine receptors (nAChRs) in the brain that modulate food intake [49,50,51,52,53,54]. This activation of nAChRs causes disruption in normal appetite regulation by increasing satiety-inducing signals [55,56,57,58] and suppressing pathways for hunger promotion [59,60,61,62,63,64]. In parallel, nicotine stimulates dopamine-mediated reward circuits, which can lead to shifts in dietary habits toward more palatable but nutrient-poor foods [57,58,65,66,67,68]. Together, these changes reduce both the quantity and quality of food intake, resulting in a persistent energy deficit. This undernutrition can suppress anabolic pathways critical for muscle maintenance, including reductions in IGF-1 and mTORC1 activity, while simultaneously increasing catabolic drivers such as myostatin and the ubiquitin–proteasome system [69]. The net outcome is reduced muscle protein synthesis, increased muscle protein breakdown, and progressive muscle loss. Over time, this imbalance contributes to a decrease in muscle mass and function, thereby increasing the risk of sarcopenia [70].

Apart from the activation of nAChRs, oxidative stress also contributes to the breakdown of muscle proteins. Rom et al. [71] have proposed a possible cellular model based on the previous literature. According to this model, cigarette smoke contains numerous toxic compounds such as ROS and RNS that, in turn, enter the bloodstream and reach the skeletal muscles, where these components increase intracellular oxidants and promote oxidative stress either directly or through activating an enzymatic source of ROS: NADPH oxidase (NOX). Increased oxidative stress will then trigger activation of the P38 MAPK signalling pathway, which contributes to muscle atrophy. Activation of this pathway can stimulate the NF-κB pathway, which is responsible for initiating the transcription of genes involved in muscle protein breakdown. A downstream effect of NF-kB is the upregulation of muscle-specific E3 ubiquitin ligases that target structural and contractile proteins such as myosin heavy chain and actin for degradation by the ubiquitin–proteasome system.

**Figure 2:**
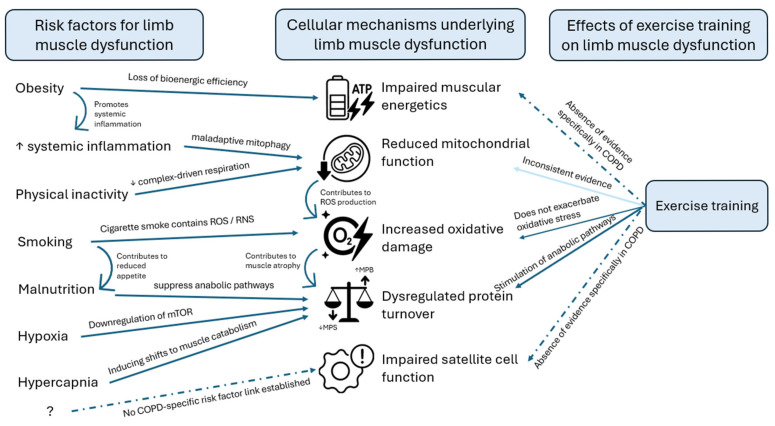
Summary of the interplay between risk factors for limb muscle dysfunction, cellular mechanisms underlying limb muscle dysfunction, and the effects of exercise training on limb muscle dysfunction. ‘?’ = not yet established risk factors that link to impaired satellite cell function.

Experimental data from human studies support this cascade: smokers display elevated oxidative stress markers in muscle and increased expression of MAFbx/atrogin-1 and MuRF1 [70,72,73,74] or increased ROS from exhaled air when compared to non-smoking healthy controls [75]. In addition, two studies investigated oxidative stress indirectly by measuring antioxidant enzyme activity and found lower antioxidant activity in smokers when compared to non-smokers [74,76]. Lastly, a study investigating mtDNA found a significant increase in mtDNA in smokers and former smokers compared to never-smokers in a dose-dependent manner [77]. Together, these findings indicate that smoking is associated with increased oxidative stress and mitochondrial alterations in skeletal muscle, highlighting a potential mechanism linking smoking to muscle dysfunction (Figure 2). While the 2014 ATS statement emphasized systemic consequences of COPD, these studies provide new evidence that smoking directly induces oxidative stress, upregulates muscle atrophy pathways, and alters mitochondrial DNA in skeletal muscle, highlighting early molecular changes that may contribute to muscle dysfunction even before the disease manifests.

### 3.2. Systemic Inflammation

Over the past few decades, studies have consistently shown that in COPD, inflammatory factors such as tumour necrosis factor α (TNF-α), interleukin-6 (IL-6), and interleukin-8 (IL-8) are elevated [78,79]. In patients with COPD, circulating TNF-α levels have been reported to be higher than in controls, and are associated with lower lean body mass [80,81] and muscle strength [82]. However, when examining muscular TNF-α levels in COPD, results have been inconclusive regarding whether these levels are increased [83], decreased [84], or unaltered [72,85]. A study by Remels et al. [86] reported that TNF-α activates hypoxia-inducible factor-1α (HIF-1α) and its target gene, vascular endothelial growth factor (VEGF), in the muscle of patients with COPD when compared to healthy matched controls. In summary, these findings indicate that inflammatory signalling contributes directly to metabolic dysregulation in COPD muscle, potentially promoting early muscle dysfunction (Figure 2). These findings expand on the 2014 ATS statement, which highlighted systemic inflammation as a contributing factor to muscle dysfunction by showing that inflammatory signalling in muscles of patients with COPD may act through HIF-1α and/or VEGF pathways, although evidence regarding local TNF-α levels remains inconsistent.

### 3.3. Hypoxia and Hypercapnia

It is known that hypoxia plays a role in skeletal muscle wasting in COPD [18,87]. Previous studies have shown that hypoxia was followed by a downregulation of the mechanistic target of rapamycin (mTOR), which is involved in the muscular anabolic pathway [88]. A more recent study in muscular myotubes demonstrated that under hypoxic conditions, the protein synthesis pathway (Akt-GSK3-β-P70S6K) was inhibited, which was associated with increased autophagy-related markers [89]. Experimental evidence from healthy participants further shows that normobaric hypoxia exacerbates inactivity-induced muscle wasting, supporting the concept that the coexistence of systemic hypoxemia and physical inactivity, as commonly observed in COPD, may accelerate skeletal muscle loss [90]. Consistent with these mechanisms, clinical data indicate that nocturnal hypoxemia is associated with lower skeletal muscle mass in COPD, with patients exhibiting nocturnal desaturation showing an additional reduction in pectoralis muscle index compared with normoxemic patients with COPD and control subjects [91]. Importantly, exercise-induced peripheral desaturation was identified as a key determinant of structural muscle adaptations, as only patients who maintained oxygen saturation during training showed significant increases in quadriceps femoris muscle cross-sectional area assessed by magnetic resonance imaging, whereas no hypertrophy was observed in those who desaturated during exercise [92]. In parallel, hypercapnia, a frequent feature in advanced COPD, has emerged as an additional systemic stressor influencing muscle metabolism [93,94]. Chronic CO_2_ retention activates AMPKα2-dependent signalling, which downregulates ribosomal biogenesis and protein synthesis while promoting proteasomal degradation, ultimately shifting muscle homeostasis toward muscle catabolism [93]. In summary, hypoxia and hypoxemia—manifesting during daily life, sleep, or exercise—impair muscle protein synthesis and limit anabolic adaptations, while chronic CO_2_ retention promotes muscle catabolism. The mechanistic link between hypercapnia, AMPK activation, and impaired muscle anabolism provides a plausible explanation for the exacerbated skeletal muscle dysfunction and poor clinical outcomes observed in CO_2_-retaining COPD phenotypes (Figure 2). While the ATS statement identified HIF and REDD1 as key mediators of hypoxia-induced muscle dysfunction, more recent studies provide insights into differences in mTOR regulation and modifications of the muscular protein synthesis pathway, thereby deepening our understanding of the impact of hypoxia and hypercapnia on muscle protein turnover.

### 3.4. Physical Inactivity and Sedentary Behaviour

One of the questions that arose from the latest update of the ATS statement in 2014 [18] was whether mitochondrial abnormalities represent disease-specific pathology or secondary effects of physical inactivity. Recently, several studies have investigated this by analysing quadriceps muscle biopsies of patients with COPD and physical-activity-matched controls [95,96], healthy controls not matched for physical activity [15,97], or (ex-)smoking populations [34]. While in one study mitochondrial respiration, expression of mitochondrial complexes I–IV, MnSOD concentrations, and markers for mitochondrial biogenesis were similar between groups [95], specific complex-driven respiration [96] and electron transport chain complexes I [34], II, III, IV, and V were significantly reduced in COPD compared to physical-activity-matched controls and healthy controls not matched for physical activity [33,34,96,97]. The results regarding mitochondrial respiration and oxidative phosphorylation were diverse but generally pointed in the same direction. There was a significant reduction in MnSOD expression in the COPD group compared to physical-activity-matched controls [96]. Both studies did find increased markers for lipid peroxidation [95,96], suggesting that patients with COPD have increased oxidative stress when compared to physical-activity-matched controls. Adami et al. [31] investigated muscular oxidative capacity in upper and lower limbs in COPD and age- and sex-matched controls, which revealed a weak correlation between the reduction in oxidative capacity in COPD and lower activity levels, suggesting a systemic impairment in mitochondrial oxidative function rather than disuse alone [31].

Although inactivity contributes to muscle wasting, COPD-related muscle dysfunction is only partly explained by disuse, with intrinsic disease mechanisms such as mitochondrial dysfunction and impaired adaptation to exercise playing a key role [95,96] (Figure 2). While the 2014 ATS statement acknowledged mitochondrial dysfunction, oxidative stress, and altered muscle protein turnover, these changes were not linked to physical inactivity or disuse. Recent evidence suggests that muscle dysfunction in COPD is not solely due to disuse or physical inactivity but also involves intrinsic mechanisms such as mitochondrial dysfunction and impaired adaptation to exercise.

### 3.5. Nutritional Status and Hormonal Changes

Malnutrition is a frequent and clinically relevant problem in patients with COPD, particularly during acute exacerbations, and is closely linked to sarcopenia, reduced respiratory and peripheral muscle function, and poorer survival [98,99]. Additionally, alterations in endocrine function can further impact skeletal muscle. A recent retrospective study found that 43% of male participants exhibited signs of energy malnutrition, defined by a respiratory quotient (RQ) of less than 0.85 [100]. Multivariate and decision-tree analyses identified reduced tidal volume and smaller erector spinae muscle cross-sectional area as independent correlates of energy malnutrition, suggesting that respiratory mechanics and muscle mass are closely linked to impaired energy balance in COPD [100]. Deficiency of specific micronutrients can also contribute to skeletal muscle dysfunction in COPD. A study by Minter et al. [101] showed significantly lower vitamin D levels in patients with COPD when compared to controls without COPD, and showed that vitamin D status was not associated with body composition or longitudinal changes in body composition. However, vitamin D insufficiency as well as low bone mineral density (BMD) were more prevalent in patients with COPD when compared to non-COPD controls [101]. In addition, a review by Russo et al. [102] concluded that vitamin D deficiency was associated with impaired muscle oxidative capacity and regenerative potential [102]. Experimental studies suggest that vitamin D supports mitochondrial function by enhancing oxidative phosphorylation and reducing oxidative stress, while activation of the vitamin D receptor promotes satellite cell activity and muscle repair [102]. In studying anabolic suppression in COPD, the insulin-like growth factor 1 (IGF-1) pathway, which plays a key role in promoting muscle growth, is of particular interest. A retrospective study of patients with COPD demonstrated significantly lower serum IGF-1 levels when compared to the control group [103]. A large population-based cohort study in community-dwelling older adults demonstrated that lower serum IGF-1 levels were associated with reduced handgrip strength and poorer physical performance, underscoring the relevance of IGF-1 for maintaining muscle function [104]. In addition to IGF-1, alterations in other anabolic hormones such as ghrelin have been reported in COPD [105]. Ghrelin, an endogenous ligand of the growth hormone secretagogue receptor, plays an important role in regulating appetite, energy balance, and muscle metabolism [106]. A recent meta-analysis demonstrated that circulating ghrelin levels are significantly elevated in patients with COPD as compared to healthy controls, particularly among those who are underweight [105]. In addition to its central effects on appetite regulation, the ghrelin receptor is also expressed in cardiac and skeletal muscle, where its activation stimulates AMPK signalling and enhances fatty acid oxidation [106]. In summary, nutrient deficiencies in COPD may also contribute to muscle dysfunction. Vitamin D insufficiency is more prevalent in COPD and has been linked to impaired muscular oxidative capacity. Additionally, lower serum IGF-1 levels are associated with reduced handgrip strength and poorer physical performance, while elevated ghrelin levels, particularly in underweight patients, may contribute to reduced appetite (Figure 2). In the 2014 ATS statement, it was acknowledged that vitamin D deficiencies were prevalent in COPD, but new evidence also shows a link between vitamin D and impaired muscle oxidative capacity and regenerative potential. Additionally, the statement discusses growth factors that included IGF-1 as part of muscle mass regulation and protein balance, but did not report specific clinical evidence showing reduced serum IGF-1 levels in patients with COPD or link these reductions to functional outcomes such as lower handgrip strength and physical performance, findings that have been described in more recent research. Lastly, in the 2014 ATS statement, plasma ghrelin was already noted to increase in underweight patients with COPD as a compensatory response to nutritional abnormalities, but ghrelin levels were not compared to healthy controls, and the mechanistic effects of ghrelin receptor signalling in cardiac muscle, AMPK activation, or fatty acid oxidation were not discussed. More recent studies have shown that circulating ghrelin is elevated in COPD, particularly in underweight patients compared with controls. Additionally, other research has shown ghrelin can activate AMPK and influence fatty acid metabolism in cardiac tissues.

### 3.6. Obesity

In the ATS statement on limb muscle dysfunction, the growing occurrence of obesity in individuals with COPD was highlighted as an emerging challenge [18]. The document emphasized that in this context, identifying low muscle mass, sarcopenia, or muscle dysfunction becomes more difficult due to the masking effect of increased body weight or BMI [18]. High BMI can paradoxically be associated with better survival, but not quality of life or physical function, particularly in advanced COPD (a phenomenon typically known as the obesity paradox) [107,108]. Notably, the obesity paradox has also been observed in apparently healthy adults and older populations and is partially explained by body composition and cardiorespiratory fitness [109]. In patients with COPD, however, these factors remain underexplored, and the independent contributions of fat mass versus lean mass to the obesity paradox, as well as its validity in individuals with sarcopenic obesity, remain to be elucidated. Recently, the European Society for Clinical Nutrition and Metabolism (ESPEN) and the European Association for the Study of Obesity (EASO) reached a consensus on the definition and diagnostic criteria for sarcopenic obesity, recognizing that obesity itself can promote loss of muscle mass and function through adipose-tissue-related metabolic disturbances such as insulin resistance [110]. Indeed, a recent study based on data from a large multicentre cohort of patients with COPD demonstrated that stratification by normal fat-free mass index (FFMI) within the same BMI category identified subgroups of patients with better lung function, exercise capacity, physical activity, health-related quality of life, and symptoms compared with their low-FFMI counterparts [111]. In a cross-sectional study involving patients with stable COPD, Persson et al. examined the relationships between thigh muscle fat infiltration, muscle bioenergetics, and systemic inflammation [112]. The study demonstrated for the first time that excessive muscle fat infiltration was associated with both systemic inflammation and reduced thigh muscle bioenergetics, suggesting a mechanistic link between inflammation and muscle fatigue mediated by fat infiltration and a loss of bioenergetic efficiency [112]. Together, these findings underscore that excess adiposity in COPD is a potential contributor to muscle dysfunction and reduced adaptive capacity. In short, these findings indicate that both low fat-free mass and excess muscle fat infiltration contribute to muscle dysfunction in COPD, linking body composition and inflammation to impaired muscle function and reduced physical capacity (Figure 2). While the 2014 ATS statement recognized that high BMI is common in COPD and may paradoxically be linked to better survival, it did not address the coexistence of obesity with low muscle mass or intramuscular fat infiltration, conditions that can exacerbate muscle dysfunction and impair physical performance in this population. Additionally, in 2014 there was still no consensus on the definition and diagnostic criteria for sarcopenic obesity, whereas such consensus is now available, thereby improving the ability to identify low muscle mass in individuals with overweight or obesity [113].

## 4. Gap 3: Effects of Exercise Training

### Molecular and Cellular Level

Exercise training is a cornerstone intervention for counteracting inactivity-induced lower-limb muscle dysfunction in COPD. In a systematic review, De Brandt et al. [107] critically examined evidence from 25 studies that investigated structural and metabolic adaptations of the vastus lateralis following different exercise-based interventions in patients with COPD [107]. Their analysis showed that training can induce partial structural and molecular remodelling of skeletal muscle [107]. These adaptations include a modest shift toward a more oxidative phenotype, reflected by a reduction in type IIb/x fibres, enlargement of fibre size, and an increased capillary-to-fibre ratio, although angiogenic responses appear blunted in hypoxemic patients [107]. In parallel, oxidative enzyme activities such as citrate synthase increase, while glycolytic enzymes remain likely unchanged [107]. Importantly, recent evidence suggests that the magnitude of these adaptations may critically depend on the progression of training volume [108]. In a post hoc analysis of a randomized trial in patients with severe COPD, those who maintained a continued increase in resistance training volume over an 8-week period demonstrated a shift toward a more oxidative muscle phenotype, with an increased proportion of type I fibres, reduced type II fibres, and higher levels of mitochondrial markers such as mitochondrial transcription factor A and hydroxyacyl-coenzyme A dehydrogenase [108]. This is further supported by biopsy data from a randomized trial in patients with severe COPD, in which resistance training induced significant increases in quadriceps citrate synthase activity, hydroxyacyl-coenzyme A dehydrogenase protein levels, and capillary-to-fibre ratio, indicating enhanced mitochondrial oxidative capacity and improved muscle microvascular structure [109]. Finally, exercise does not appear to exacerbate oxidative stress or inflammation, although antioxidant responses are often attenuated in COPD when compared to healthy controls [107]. Consistent with this, biopsy studies in patients with COPD have shown no evidence of muscular inflammation at baseline and no induction of muscle inflammatory responses following either endurance or resistance training [114]. Instead, both training modalities increased muscle antioxidant capacity, as reflected by upregulation of SOD2, without changes in muscular NOX content or muscle macrophage infiltration [114]. In addition, a randomized controlled trial combining high-intensity interval training with power training demonstrated a significant reduction in systemic oxidative stress, as reflected by decreased plasma protein carbonylation [115]. Follow-up data from the same cohort indicate that while improvements in muscle thickness, power, and physical function were partly preserved up to 10 months after training cessation, the exercise-induced reductions in systemic oxidative stress were not maintained, suggesting that redox adaptations may be more transient than structural muscle adaptations in COPD [116].

In summary, exercise training partially reverses lower-limb muscle dysfunction in COPD by promoting structural and molecular adaptations, improving oxidative capacity, and stimulating anabolic regulators, though responses are often blunted compared to healthy individuals (Figure 2). The 2014 ATS statement highlighted the trainability of limb muscles and improvements in strength and oxidative capacity with exercise, but did not address recent evidence showing that COPD exhibits blunted anabolic signalling, incomplete reversal of fibre type shifts, impaired angiogenic responses, and unchanged oxidative stress and inflammation with training, suggesting a partially blunted response to training, potentially due to disease-specific alterations in anabolic signalling, fibre composition, and angiogenic capacity.

Building on these findings, when examining the effects of exercise training programmes, it was found that training-induced energy production pathways, such as oxidative phosphorylation and amino acid biosynthesis, were not activated in COPD [107]. After training, the ATP synthesis rate and phosphate/oxygen (ATP/O) ratio increased in the COPD group. In both groups, mitochondrial biogenesis and mitochondrial fusion capacity were enhanced. These results indicate altered mitochondrial function and impaired adaptation to exercise training in COPD, independent of disuse, suggesting that disease-specific mechanisms underlie exercise intolerance. One study also examined the effects of exercise training and showed improvements in mitochondrial function in physical-activity-matched controls, characterized by increased respiration, ATP synthesis, and expression of complexes I, III, and IV, along with MnSOD and TFAM. In patients with COPD, only ATP synthesis and ATP/O ratio improved, and lipid peroxidation decreased [95]. When examining the effects of exercise training [95], it was observed that in the healthy controls, mitochondrial respiration, ATP synthesis rate, and the expression of complexes I, III, and IV increased. In contrast, in the COPD group, there were no changes in mitochondrial respiration or the expression of complexes I–IV. Lastly, a study investigating electrical pulse stimulation (EPS) in COPD and healthy myotubes reported that expression of mTOR was not induced, and expression of TFAM and COX1 tended to be reduced in COPD compared to healthy myotubes after EPS [117]. In summary, exercise-induced mitochondrial and energy-production pathways appear impaired in COPD, with evidence of altered oxidative metabolism, greater reliance on anaerobic pathways and blunted mitochondrial adaptations compared to healthy controls. Overall, these findings suggest a reduced capacity for muscular bioenergetic adaptation in response to exercise in COPD (Figure 2). The 2014 ATS statement recognized baseline mitochondrial abnormalities and improvements in oxidative enzyme activity with training, but more recent evidence indicates that exercise-induced mitochondrial and energy-producing adaptations are blunted in COPD, with altered oxidative metabolism and greater reliance on anaerobic pathways, suggesting reduced capacity for adaptations in response to exercise.

## 5. Conclusions

This review has several strengths and limitations. Firstly, in this study, no systematic search strategy was applied, which could increase the risk of selection bias. Additionally, no quantitative effect was estimated, and pooled analyses were not conducted. Moreover, despite the ATS statement of 2014 emphasizing the importance of appropriate control groups, considerable heterogeneity in study design, populations, and outcome measures remains, often hampering meaningful comparisons between studies, a limitation which is also observed in the present review. Another limitation is that the present review focused primarily on the effects of exercise training on molecular and cellular mechanisms underlying skeletal muscle plasticity in COPD, rather than on body composition and functional outcomes. These clinically relevant aspects have been addressed elsewhere and were therefore considered beyond the scope of the current review [118]. Strengths of the current review were that firstly, a broad integration of molecular, cellular, and functional evidence could be included, therefore enabling contextual interpretation of recently emerged research. Additionally, the current review facilitates new hypothesis generation and identification for future research directions in the field of lower-limb muscle dysfunction in COPD.

Over the past decade, substantial progress has been made in understanding the cellular and molecular mechanisms that contribute to limb muscle dysfunction in COPD. The current review highlighted these new findings by focusing on cellular mechanisms such as altered muscle protein synthesis and muscle protein breakdown, impaired mitochondrial function, increased oxidative damage, altered muscular energetics, and impaired function of satellite cells. Furthermore, evidence on risk factors for the onset of limb muscle dysfunction such as smoking, systemic inflammation, hypoxemia and/or hypercapnia, physical inactivity, nutritional status, hormonal changes, and obesity were highlighted. Lastly, the effects of exercise training on limb muscle dysfunction were discussed. From the evidence of the last decade, it can be concluded that limb muscle dysfunction is a multifactorial process driven by both intrinsic alterations to the muscle and extra-pulmonary influences, thereby reinforcing the need for integrated therapeutic strategies.

## 6. Suggestions for Future Research

In this narrative review, we revisited several research gaps previously identified in the ATS statement [18]; however, when integrating the newer literature, additional and evolving gaps in our understanding of limb muscle dysfunction have become evident. Despite evidence of disrupted anabolic and catabolic signalling in skeletal muscle of patients with COPD, it remains unclear how these molecular abnormalities—together with impairments in mitochondrial function, muscular energetics, and satellite cell function—collectively drive changes in muscle mass and strength. Addressing this gap will require integrative studies that connect molecular and cellular mechanisms to clinically relevant outcomes such as body composition and exercise capacity. Additionally, although smoking has been identified as a key contributor to early muscle dysfunction, the long-term trajectory of these effects remains unclear. Longitudinal studies are needed to determine how smoking-related muscle dysfunction progresses over time and whether early dysfunction may predict later clinical outcomes. Moreover, while several risk factors for limb muscle dysfunction have been identified in COPD, the long-term impact of these factors remains poorly understood. In particular, the growing prevalence of obesity in COPD highlights the need for longitudinal studies to examine how these risk factors contribute to limb muscle dysfunction over time. Furthermore, additional research is needed to explore other potential contributors that have not yet been investigated. Lastly, recent studies have investigated cellular and mitochondrial mechanisms before and after exercise; however, these studies have not directly linked these mechanistic changes to exercise capacity or muscular strength. Additional research is needed on cellular and mitochondrial adaptations in response to exercise and their translation to exercise performance and muscular strength.

## Data Availability

No new data were created or analysed in this study. Data sharing is not applicable to this article.

## Abbreviations

The following abbreviations are used in this manuscript:

6MWT	6-min walk test
ADP	Adenosine diphosphate
Akt1	Protein kinase B alpha
AMPK	AMP-activated protein kinase
ATP	Adenosine triphosphate
ATP/O ratio	ATP per oxygen ratio
ATS	American Thoracic Society
BNIP3	BCL2/adenovirus E1B 19 kDA-interacting protein 3
COPD	Chronic obstructive pulmonary disease
CRP	C-reactive protein
EPS	Electrical pulse stimulation
FFMI	Fat-free mass index
FOXO	Forkhead Box O
FUNDC1	FUN14 domain-containing protein 1
GOLD	Global Initiative for Chronic Obstructive Lung Disease
H_2_O_2_	Hydrogen peroxide
HIF-1α	Hypoxia-inducible factor-1 alpha
HNE	4-hydroxynonenal
IDH2	Isocitrate dehydrogenase 2
IGF-1	Insulin-like growth factor 1
IL-6	Interleukin 6
IL-8	Interleukin 8
LC3	Microtubule-associated protein 1A/1B-light chain 3
LC3BII/I	Ratio of LC3B-II to LC3B-1
MAFbc	Muscle atrophy F-box
MnSOD	Manganese superoxide dismutase
MHC	Myosin heavy chain
miRs	Micro-ribonucleic acid
mtDNA	Mitochondrial DNA
MuRF-1	Muscle RING finger-1
Myf5	Myogenic factor 5
MyHC-I	Myosin heavy chain type I
MyoD	Myoblast determination protein
nACHs	Nicotinic acetylcholine receptors
NF-kB	Nuclear factor kappa B
NMES	Neuromuscular electrical stimulation
NOX	NADPH oxidase
OHdG	8-Hydroxy-2′-deoxyguanosine
P38 MAPK	P38 mitogen-activated protein kinase
Pax7	Paired box protein 7
PCr	Phosphocreatine
P-4E-BP-1	Phosphorylated 4E-binding protein 1
PINK1	PTEN-induced putative kinase 1
p-ULK1	Phosphorylated Unc-54-like kinase 1
RNS	Reactive nitrogen species
ROS	Reactive oxygen species
SIRT1	Sirtuin 1
SOD2	Superoxide dismutase 2 (MnSOD)
TCA	Tricarboxylic acid cycle
TFAM	Mitochondrial transcription factor A
TNF-α	Tumour necrosis factor alpha
VEGF	Vascular endothelial growth factor
